# Complete Genome Sequence of Lytic Bacteriophage VPUSM 8 against O1 El Tor Inaba *Vibrio cholerae*

**DOI:** 10.1128/genomeA.00073-17

**Published:** 2017-05-25

**Authors:** Ali Al-Fendi, Rafidah Hanim Shueb, Phiaw Chong Foo, Manickam Ravichandran, Chan Yean Yean

**Affiliations:** a Department of Medical Microbiology and Parasitology, School of Medical Sciences, Universiti Sains Malaysia, Health Campus, Kubang Kerian, Kelantan, Malaysia; b Department of Biology, Faculty of Science, Cihan University Campus-Erbil, Kurdistan Region, Iraq; c School of Health Sciences, Universiti Sains Malaysia, Health Campus, Kubang Kerian, Kelantan, Malaysia; d Department of Biotechnology, Faculty of Applied Sciences, AIMST University, Semeling, Bedong, Kedah, Malaysia; e Institute for Research in Molecular Medicine, Universiti Sains Malaysia, Health Campus, Kubang Kerian, Kelantan, Malaysia

## Abstract

The complete genome sequence of bacteriophage VPUSM 8 against O1 El Tor Inaba *Vibrio cholerae* is reported here. The isolated VPUSM 8 has potential use in future phage therapy or as a biocontrol agent for the prevention and treatment of cholera.

## GENOME ANNOUNCEMENT

A lytic bacteriophage, VPUSM 8, which acts against *Vibrio cholerae* O1 El Tor Inaba strain, was isolated from sewage water in Kelantan, Malaysia. A detailed investigation of the VPUSM 8 genome could shed further light on the role of bacteriophage in bacterial population dynamics in the environment and possible development of the bacteriophage as a biological control agent in areas that are endemic for cholera (1).

Vibriophage VPUSM 8 was propagated in the *V. cholerae* O1 El Tor Inaba strain. Bacteriophage genomic DNA was extracted using previously described methods (1), and the whole genome was sequenced using the PGM Ion Torrent sequencing technology with 316 D Chip (Life Technologies, Inc.) at AITBiotech, Singapore. Sequence reads were processed on the Ion Torrent server to remove adapter sequences, and a quality score was assigned. Reads were assembled in Mimicking Intelligent Read Assembly (MIRA) version 3.9.9 using the *de novo* method (2, 3). Coding sequences (CDSs) within the VPUSM 8 genome were allocated using Vector NTI Advance and CDS Finder software from the National Center for Biotechnology Information (NCBI) using bacterial start and stop codon options (http://www.ncbi.nlm.nih.gov/gorf/gorf.html).

Morphological analysis by transmission electron microscopy showed that bacteriophage VPUSM 8 belongs to the *Myoviridae* family (1, 4), while the genome sequence suggests that it is a P2-like bacteriophage (5). The genome of VPUSM 8 was 34,145 bp, with a G+C content of 48.92% and 43 identified CDSs. These 43 CDSs occupy 93.25% of the total genome and vary in length from 209 bp (CDS 25) to 2,504 bp (Rep). The bacteriophage VPUSM 8 contains 6.75% noncoding sequence. Four gene pairs overlapped between 10 and 91 nucleotides (CDS14-CDS15, CDS21-CDS22, CDS30-CDS31, and CDS36-CDS37). From the total predicted putative genes, 34 genes are transcribed from the same strand, and only 9 genes (Int, Glo, CI, and CDSs 2, 11, 13, 14, 15, and 16) are transcribed from the reverse strand. Out of 43 CDSs mentioned above, 41 potential gene sequences are with the ATG initiation codon, while another 2 sequences are with GTG. Three rho-independent transcription terminators, seven promoters, and other additional elements were identified based on homology to K139 phage (accession no. AF125163.2), with which VPUSM 8 shares 98% nucleotide identity (6, 7). However, some VPUSM 8 CDSs that are presumably involved in phage replication, the lysis-lysogeny region, and capsid and tail formation (CDSs CI, CII, Rep, 15, and 34) show differences at the nucleotide and amino acid levels (1). Additionally, CDS 22 and CDS 23 in K139 phage are present only in one CDS, the CDS 22, in VPUSM 8.

### Accession number(s).

The complete genome of lytic vibriophage VPUSM 8 was deposited in GenBank under the accession no. KF361475.1.

## References

[1] Al-Fendi A, Shueb RH, Ravichandran M, Yean CY 2014 Isolation and characterization of lytic vibriophage against *Vibrio cholerae* O1 from environmental water samples in Kelantan, Malaysia. J Basic Microbiol 54:1036–1043. doi:10.1002/jobm.201300458. 24532381

[2] Gordon D, Green P 2013 Consed: a graphical editor for next-generation sequencing. Bioinformatics 29:2936–2937. doi:10.1093/bioinformatics/btt515. 23995391 PMC3810858

[3] Li R, Zhu H, Ruan J, Qian W, Fang X, Shi Z, Li Y, Li S, Shan G, Kristiansen K, Li S, Yang H, Wang J, Wang J 2010 *De novo* assembly of human genomes with massively parallel short read sequencing. Genome Res 20:265–272. doi:10.1101/gr.097261.109. 20019144 PMC2813482

[4] Ackermann HW 2009 Phage classification and characterization. Methods Mol Biol 501:127–140. doi:10.1007/978-1-60327-164-6_13. 19066817

[5] Talledo M, Rivera IN, Lipp EK, Neale A, Karaolis D, Huq A, Colwell RR 2003 Characterization of a *Vibrio cholerae* phage isolated from the coastal water of Peru. Environ Microbiol 5:350–354. doi:10.1046/j.1462-2920.2003.00411.x. 12713461

[6] Reidl J, Mekalanos JJ 1995 Characterization of *Vibrio cholerae* bacteriophage K139 and use of a novel mini-transposon to identify a phage-encoded virulence factor. Mol Microbiol 18:685–701. doi:10.1111/j.1365-2958.1995.mmi_18040685.x. 8817491

[7] Kapfhammer D, Blass J, Evers S, Reidl J 2002 *Vibrio cholerae* phage K139: complete genome sequence and comparative genomics of related phages. J Bacteriol 184:6592–6601. doi:10.1128/JB.184.23.6592-6601.2002. 12426348 PMC135448

